# Making hospital shops healthier: evaluating the implementation of a mandatory standard for limiting food products and promotions in hospital retail outlets

**DOI:** 10.1186/s12889-020-8242-7

**Published:** 2020-01-30

**Authors:** Martine Stead, Douglas Eadie, Jennifer McKell, Leigh Sparks, Andy MacGregor, Annie S. Anderson

**Affiliations:** 1 Institute for Social Marketing and Health, Faculty of Health Sciences and Sport, University of Stirling, Stirling, UK; 2 Institute for Retail Studies, Stirling Management School, University of Stirling, Stirling, UK; 3 ScotCen, Edinburgh, UK; 4 Centre for Public Health Nutrition Research, School of Medicine, University of Dundee, Dundee, Scotland

**Keywords:** Retail, Hospitals, Promotions, Mandatory regulation, Implementation, Evaluation, Patients, Mixed methods

## Abstract

**Background:**

The range of products stocked and their promotions in food retail outlets in healthcare settings can affect food choices by staff, patients and visitors. The innovative Scottish Healthcare Retail Standard (HRS) is a national mandatory scheme requiring all hospital food retail outlets to change the balance of food products stocked and their promotion to comply with nutritional criteria and promotional restrictions. The aim is to facilitate healthier food choices in healthcare settings. This study examined the implementation of HRS and the impact on foods stocked and promoted.

**Methods:**

The study aimed to examine implementation process and changes to the retail environment in relation to food promotions and choice. A sample of hospital retail outlets (*n* = 17) including shops and trolley services were surveyed using a mixed methods design comprising: (a) structured observational audits of stock, layout and promotions (with a specific focus on chocolate and fruit product lines), and (b) face-to-face, semi-structured interviews with the shop manager or nominated members of staff (*n* = 32). Data were collected at Wave 1 (2016), at the beginning and during the early stages of HRS implementation; and Wave 2, 12 months later, after the HRS implementation deadline.

**Results:**

All outlets, both commercial and not-for-profit, in the sample successfully implemented HRS. Implementation was reported to be more challenging by independent shop managers compared to chain store staff. Retail managers identified areas where more implementation guidance and support could have been provided. The number of chocolate product lines and promotions reduced substantially between Waves 1 and 2, but with no substantial increase in fruit product lines and promotions. Despite initial negative expectations of HRS’s impact, managers identified some opportunities in the scheme and positive changes in the supply chain.

**Conclusions:**

Positive changes in food retail outlets occurred after hospital shops were required to implement HRS. By creating a consistent approach across hospital shops in Scotland, HRS changed the food retail environment for hospital staff, visitors and patients. HRS provides a regulatory template and implementation learning points for influencing retail environments in other jurisdictions and settings.

## Background

Twenty-nine percent of Scottish adults are obese [1], increasing risk for diabetes, cardiovascular disease and cancer [2]. Despite numerous health and nutrition education programmes from the public sector, voluntary sector and celebrity chefs, there has been little change in diet in Scotland over the last decade and little progress towards meeting the Scottish Dietary Goals [3], notably among households in the areas of most deprivation where obesity levels in women and children are highest [1].

Reflecting global concern about the role of diet and obesity in the development of non-communicable diseases, public health action in Scotland has turned to issues of access, pricing and marketing of energy-dense foods and drinks [4]. There is growing interest in the role of the food retail environment in shaping consumer preferences and behaviour [5]. Calls have been made for interventions including regulations and economic incentives to tackle ‘obesogenic’ retail environments [6, 7]. Most population-based retail-focused interventions have focused on information, exhortation and education (e.g. traffic lights labelling) or voluntary reduction schemes (e.g. portion size), with little effect on caloric intake. For example, the English ‘Public Health Responsibility Deal’ programme (a public-private partnership with voluntary agreements) has been criticised [8] for failing to incorporate food pricing strategies, restrictions on marketing, and reducing sugar intake in its attempts to change diet. Whilst some retail intervention studies have reported using food pricing [9], marketing approaches [10, 11] and placement strategies [12], few have produced significant or sustainable changes in food purchases. Most interventions have taken place *within* the existing retail environment – i.e. increasing consumers’ healthy choices – rather than transforming this environment to tackle the “*excess availability and affordability”* of food ([13], p348).

The food retail environment includes healthcare settings. Food retail outlets are present in many hospital buildings and campuses, and are used widely by staff, patients and visitors. Although there is widespread recognition that health promotion is central to the provision of healthcare [14], the potential for healthcare systems to promote and facilitate appropriate food choices and to act as an exemplar for other sectors is underdeveloped. While various initiatives have been implemented both in Scotland and other countries to increase the healthiness of catering in hospitals [15, 16], these initiatives have not included retail outlets, resulting in inconsistent messages and practices (such as serving healthy food in the canteen but selling chocolate on promotion in the hospital shop). This is a particular concern given rising rates of obesity among healthcare staff in the UK [17, 18] and the potential of worksites to improve lifestyles [19–23].

### The healthcare retail standard

In 2010, the Scottish Government set out a strategy for healthy weight in Scotland which outlined a range of preventative actions, including energy intake, food product reformulation, portion sizes, pricing, packaging, and advertising, with the aim of reducing obesity levels [24]. As part of this strategy, the Healthcare Retail Standard (HRS) was introduced in 2015, comprising a set of mandatory requirements for retail outlets in NHS healthcare premises in Scotland [25]. HRS required that a substantial and specified proportion of food and drinks on sale must meet nutritional criteria, and that only products meeting nutritional criteria could be promoted (Table 1).

**Table 1:** Healthcare Retail Standard: summary of requirements

HRS element	Summary of requirements	Notes
Type of outlet to which HRS applies	Retail outlets – those where food is not prepared on-site but is ready for immediate purchase and consumption. Examples include a convenience store, newsagent, mobile or pop-up store or trolley service. Mixed outlets – those which offer a combination of catering and retail provision. Mixed outlets in healthcare buildings should comply with both the HRS and the existing Healthy Living Award Plus scheme for catering outlets.	The Healthy Living Award Plus scheme is a reward scheme for catering establishments in Scotland which demonstrate a high level of commitment to supporting healthy eating. Similar to HRS, establishments must meet criteria concerning the balance of the product range and avoiding promotion of less healthy items. http://www.healthylivingaward.co.uk/caterers/the-plus-award
Provision criteria	Retail outlets should stock a range of food items that are not high in fat, salt and sugar. At least 50% of food items and at least 70% of drinks must meet specified nutrition criteria. Water is not included (ie. 70% of drinks excluding water have to meet specified nutrition criteria).	Nutrition criteria are set out here: http://www.gov.scot/Publications/2016/10/5243/7
Promotions criteria (individual products)	Only food items/products that meet specified nutrition criteria can be promoted. Promotions are defined as: ‘a mechanic or action used to induce consumers to purchase a product which was otherwise not intended to be purchased’, including: • price reductions, • multi-buys, • prominently positioned displays (e.g. at till points, at the outlet entrance, in dump bins, at gondola ends and at the queue management system • and others, including up-selling (verbal suggestion by the till assistant) (SGF^15^).	Originally all price-marked packs (packs with the price printed prominently on the packaging) were defined as promotions and therefore not permitted for products not meeting specified nutrition criteria. The HRS rules were amended following feedback from retailers that some items were only available in such packaging. After considering different product sizes, the Scottish Government agreed to allow price-marked packs if the price-marking covered less than 25% of the pack face.
Promotions Criteria for ‘Meal Deals’: promotional bundles offering a sandwich (or similar), snack and drink for a fixed price.	Only products which are permitted to be promoted can be included in a meal deal. Meal deals should: ◦ be based around starchy carbohydrates such as bread, potatoes, rice and pasta; ◦ contain a portion of fruit and/or vegetables; and ◦ items included should not be high in fats, salt or sugars.	Originally only fruit was allowed as the snack item in a meal deal. However, a subsequent increase observed in sales of crisps (and decline in sales of healthier alternatives) led to amendment of the meal deal rules to permit the inclusion of baked crisps.
Monitoring and compliance	All retail outlets run in-house (ie. by the NHS) and by the voluntary sector must comply. NHS Boards are required to have HRS as a mandatory condition of any contract negotiated with a commercial retail outlet. A monitoring scheme is run by Scottish Grocers’ Federation (SGF), the trade association for the retail convenience sector in Scotland. SGF provides guidance to retailers on how to meet the HRS requirements and conducts inspections to assess initial compliance. Quality assurance inspections will then be conducted at least every 2 years.	

HRS applied to all retail outlets in Scottish healthcare premises, including outlets operated by major national retail groups [26]. Hospitals have contracts with retail outlets, and adherence to HRS was made a condition of contract renewal; this provided a mechanism for enforcement. An 18-month implementation period was established, October 2015 to March 2017, with all outlets expected to pass a compliance inspection by the end of March 2017. HRS represents to our knowledge the first national-level mandatory scheme in hospital retail outlets that addresses access, promotions and product range, seeking not only to increase healthy choices but also to reduce or remove unhealthy products and promotions. It provides a distinctive contribution to the evidence base on food retail interventions. In this paper we examine retailers’ experiences of implementing HRS and the impact of implementation on food and drink product range and promotions.

## Methods

We conducted a mixed methods study of HRS implementation in a sample of hospital retail outlets. This comprised: 1. a structured observational audit of stock, layout and promotions and 2. face-to-face, semi-structured interviews with the shop manager or nominated member of staff. Data collection was conducted at two waves: Wave 1, during the early stages of HRS implementation (July to November 2016), and Wave 2, between July and November 2017, after the HRS implementation deadline of March 2017.

### Sample

From a list of hospital food retail outlets provided by NHS Health Scotland, the investigators selected a purposive sample (*n* = 13) designed to achieve heterogeneity in terms of the following variables:

- type of management (commercial/not-for profit; independent/part of retail group or large charity)
- health board area (outlets were recruited from six of Scotland’s 14 health boards)
- hospital location and catchment area (both urban and mixed urban-rural areas were included; rural outlets were excluded because they were generally smaller in terms of their size, food and drink range and customer base)
- progress towards HRS compliance at baseline (some of the outlets had partially implemented HRS at Wave 1, whereas others had not yet started).

Retail owners or managers were contacted (following permission from their head office or directly, if the shop was independently operated) with a letter and study information sheet, and then followed up by telephone or email to explain the study, answer any queries and set up an appointment to visit. Written consent was obtained at the start of each fieldwork visit. Four trolley services (mobile carts which are taken around hospital wards, usually by volunteers, with a range of food, drink and small gifts, primarily for patient use) were additionally surveyed. These were selected from those shops in the sample which operated trolley services and where trolleys were sufficiently stocked, at the time of the Wave 1 fieldwork visit, to represent a typical service offering.

The final sample comprised five shops operated by commercial companies (three operated by a large national retailer, one operated by another large national retailer, and one independent), eight shops operated by not-for-profit^1^ organisations (six operated by a large national charity, one operated by the local NHS, and one operated by a local voluntary organisation), and four trolley services (all operated by a large national charity), operated by shops 5, 8, 9 and 13 (Table 2). The sample represented 18% of the 72 retail outlets and 10% of the 39 trolley services to which HRS applied [27].

**Table 2:** Characteristics of the shops

Shop	Type of hospital	Characteristics of outlet
1	Very large hospital serving large city population	Commercial: Operated by large national retailer
2	Very large hospital serving large city population	Commercial: Operated by large national retailer
3	Very large hospital, urban area	Commercial: Operated by large national retailer
4	Very large hospital, urban area	Commercial: Independently owned commercial retail outlet
5	Medium sized hospital, urban area with large rural and semi-rural catchment	Not-for-profit: Operated by local hospital volunteers
6	Large hospital, city centre location	Commercial: Operated by large national retailer
7	Large hospital, city location with large rural and semi-rural catchment	Not-for-profit: Operated by local NHS catering service
8	Large hospital, city location with large rural and semi-rural catchment	Not-for-profit: Operated by large national charity
9	Large hospital, urban area	Not-for-profit: Operated by large national charity
10	Large hospital, city centre location	Not-for-profit: Operated by large national charity
11	Small, non-acute, specialist hospital, city location	Not-for-profit: Operated by large national charity
12	Medium sized acute hospital, serving a large town and surrounding area	Not-for-profit: Operated by large national charity
13	Medium sized hospital, urban area	Not-for-profit: Operated by large national charity

In-depth one-to-one and paired interviews were conducted by three experienced qualitative researchers (two female and one male: MS, JM and DE) with a range of retail staff (*n* = 32: 16 per wave). The majority (*n* = 24) were local managers. Interviews were also conducted with four nominated supervisors, two regional managers, one business proprietor and one assistant. Ten Wave 1 interviewees participated again at Wave 2, most of whom were managers. Some Wave 1 interviewees could not participate at Wave 2 due to unavailability at the time of fieldwork or having moved to a different job.

### Observational audit

Observational audit protocols were developed and piloted in shops and for trolley services selling similar products in non-hospital settings (see Additional files 1, 2, 3, 4 and 5). The final protocols recorded a range of measures relating to outlet size, layout, product range, promotions and advertising materials. In this paper we focus on measures relating to two product categories, chocolate and fresh fruit. These product categories were selected as exemplar ‘snack’ products, in the less healthy, non-compliant category (products not permitted to be promoted under HRS) and in the healthy, compliant category (products which could be stocked and promoted with no restrictions under HRS). Table 3 outlines how the two categories were defined.

**Table 3:** Definitions of chocolate and fruit

Product category	Definition
Chocolate	Solid blocks of chocolate, blocks with added ingredients, such as fruit and nuts, chocolate eggs, confectionery that contains chocolate as the main ingredient, chocolate-covered confectionery in bags, rolls and tubes. (definition based on Mintel categories [28]).
Fruit	Fresh fruit: Sold loose or pre-packed Fresh fruit salad/fruit pots (Dried fruit products were excluded because they did not meet the HRS criteria for products which could be promoted.

Snack products such as crisps, cereal bars and non-chocolate confectionery were not selected for examination because the existence of ‘healthier’ variants of each (e.g. sugar free confectionery, baked crisps) would have made direct comparison less reliable. The following measures are reported:

- Number of relevant products on display. These were counted at Stock-Keeping Unit (SKU) level, i.e. distinct product lines, rather than number of product facings.
- Number of promotions for relevant products including the following types:
  - Product displays (including free-standing merchandising units, either temporary or permanent, and temporary product stacks designed to feature a particular brand or product).
  - Price-marked packs (PMPs) (products with the price printed in large type on the pack/wrapper designed to catch attention).
  - Multi-buys or quantity discounts (offers such as ‘3 for the price of 2’).
  - Advertising (posters, stands, leaflets, electronic screens, shelf-edge signage).
  - Other (e.g. verbal promotions by till staff).

Multiple instances of the same promotion applied to the same product were counted once only.

### In-depth interviews

Interviews were conducted using a semi-structured discussion guide developed for the study (see Additional files 1, 2, 3, 4 and 5). The guide explored: awareness, understanding of and attitudes towards HRS; the implementation process; barriers and facilitators to implementation; perceived impact on business and customer response; and any unforeseen consequences. All but one of the interviews were audio-recorded with participants’ consent and transcribed verbatim for analysis (one manager declined audio-recording and instead notes were taken, both during and immediately after the interview). Interviews ranged in duration from 17 min to 1 h 10 min with the majority lasting more than 40 min, and were conducted in or near outlets, in a nearby administrative office, café, canteen or hospital foyer. Interviewees were offered a small incentive of £10 in cash at each wave as a thank you for their input and to compensate them for any inconvenience caused.

### Data analysis

Observational audit data were entered into an Excel spreadsheet to enable comparison of the two waves using descriptive statistics; no statistical tests were conducted as the sample size was too small. Changes to store layout were recorded using photographs and hand drawn diagrams generated by the researchers at each wave. All textual data including transcripts and field notes were coded thematically, by the same researchers who conducted the interviews, using a thematic framework approach, facilitated by the management and organisation of data in tables. The coding framework used drew on themes identified from the interview guide as well as themes arising from the data. Interview and observation data were analysed together where appropriate and triangulated to assess for consistency. Ethical review and approval were provided by the University of Dundee Research Ethics Committee.

## Results

All but one shop had achieved compliance by the March 2017 deadline (after making some amendments, the remaining shop passed a subsequent inspection). Compliance levels were broadly consistent with those across the total population of hospital shops to which HRS applied, with 54 of 72 shops achieving compliance by the March 2017 deadline, and 70 by May/June of the same year (2 outlets were not assessed) [27].

### Observational audit findings

#### Changes in product range: chocolate and fruit

The mean number of chocolate confectionery SKUs observed on display per shop reduced from 60 products (standard deviation (SD) =36) (range 10–126) at Wave 1 to 29 (SD =12) (range 12–50) at Wave 2. There was no change in the number of fruit products on display in the outlets between Wave 1 and Wave 2, a mean of 10 per wave (Wave 1 SD = 13, range 3–51; Wave 2 SD = 14, range 4–56). Commercial retail outlets stocked slightly more chocolate and fruit SKUs than not-for-profit outlets at Wave 1, but experienced a similar level of reduction in chocolate SKUs and of increase in fruit SKUs at Wave 2, compared with not-for-profit outlets. The four trolley services saw a decrease in the mean number of chocolate products on display at Wave 2 (from 15 to 12) and limited change in the mean number of fruit SKUs, from 2 to 3.

#### Changes in promotions

The total number of promotions observed for chocolate across all 13 shops was 166 at Wave 1, of which 95 (57%) were observed in the five commercial shops, and 71 (43%) were observed in the eight not-for-profit shops (Table 4). Just over a third (36%) consisted of multi-buys/quantity discounts, and just under a third (32%) were PMPs. Commercial outlets made proportionately more use of multi-buys/quantity discounts (56% of all chocolate promotions in these outlets), while not-for-profit outlets made proportionately more use of PMPs (62% of all promotions in these outlets). At Wave 2, the total number of promotions observed had fallen substantially to 38. The vast majority (92%) of the remaining promotions observed at Wave 2 were PMPs which were permitted under HRS as the price-marking covered less than 25% of the face (see Table 1). Commercial outlets had made relatively little use of PMPs at Wave 1, with only seven instances observed, but slightly increased their usage of these at Wave 2 (20 instances observed), once other forms of promotions had been prohibited. On the trolley services, the mean number of promotions for chocolate decreased slightly from 6 at Wave 1 to 4 at Wave 2 (all PMPs on one of the four trolleys; no other promotions were observed).

**Table 4:** Chocolate and fruit promotions, Wave 1 vs. Wave 2

	Total N	Product displays		PMPs		Multi-buys/ quantity discounts		Advertising		Other	
		N	*%*	N	*%*	N	*%*	N	*%*	N	*%*
Chocolate											
Wave 1 all shops	166	25	*15*	51	*32*	60	*36*	21	*13*	9	*5*
Commercial	95	12	*13*	7	*7*	53	*56*	15	*16*	8 (8%)	*8*
Not-for-profit	71	13	*18*	44	*62*	7	*10*	6	*8*	1	*1*
Wave 2 all shops	38	3	*8*	35	*92*^a^	–		–		–	
Commercial	*23*	*3*	*13*	*20*	*87*^*a*^	*–*		*–*		*–*	
Not-for-profit	*15*	*–*		*15* ^*a*^	*100*^*a*^	*–*		*–*		*–*	
Fruit											
Wave 1 all shops	52	11	*21*	10	*19*	18	*35*	13	*25*	–	
Commercial	*24*	*3*	*13*	*9*	*38*	*10*	*42*	*2*	*8*	*–*	
Not-for-profit	*28*	*8*	*29*	*1*	*4*	*8*	*29*	*11*	*39*	*–*	
Wave 2 all shops	69	14	*20*	9	*13*	12	*17*	31	*45*	3	*4*
Commercial	*29*	*5*	*17*	*8*	*28*	*6*	*21*	*9*	*31*	*1*^b^	*3*
Not-for-profit	*40*	*9*	*23*	*1*	*3*	*6*	*15*	*22*	*55*	*2*^c^	*5*

^a^All PMPs were compliant with HRS rules

^b^Art work

^c^Verbal reinforcement of multi-buy offer, discounted fruit at till

The total number of fruit promotions observed across all 13 shops increased slightly, from 52 at Wave 1 to 69 at Wave 2 following HRS implementation. Although all forms of promotion were permitted for fruit, the majority of the increase was accounted for by advertising materials (many of which were provided free to outlets by Scottish Grocers Federation (SGF)), with relatively little increase in use of other forms of promotion; indeed, observed use of multi-buys/quantity discounts decreased, from 18 instances at Wave 1 to 12 instances at Wave 2. On the trolley services, there was no change in the number of fruit promotions, with only one multi-buy offer observed on one trolley at both waves.

### Interview findings

#### Rebalancing the product range

HRS involved a substantial reconfiguration of the product range to achieve at least 50% compliance with nutrition criteria. Typically, managers referred to ‘being allowed’ a proportion of non-compliant items, and juggled the product range to increase this ‘allowance’ by introducing new product categories such as grocery items, or sugar-free confectionery: ‘Because of the size of my shop, I can take every single kind of sugar-free sweets …. that then enabled me to have slightly more [non-compliant sweets]’ (Outlet 12, Wave 2). Outlets operated by the national charity applied the 50/50 rule within each type of product – i.e. at least 50% of crisps had to be compliant – resulting in considerable simplification of the product range. Managers found this a useful way to emphasise the requirements clearly to staff and volunteers, facilitating adherence and reducing errors: ‘So, if they [put on display] five bars of non-compliant chocolate, they need to balance it out with five compliant healthy snacks’ (Outlet 10, Wave 2). In some outlets, managers sought to overcompensate on the 50/50 criterion (for example, aiming for a 60/40 balance in favour of compliant products) to avoid the outlet inadvertently slipping into non-compliance prior to an inspection. Increasing the compliant product range required the identification of new suppliers, and these arrangements took time to establish, with some managers describing problems with reliability and availability, and with negotiating small orders for ‘healthier’ products which were less popular sellers. Most shops had to reconfigure their layouts and planograms to adapt to the reduced confectionery range and increase in other product categories, in some cases also having to make physical changes to shelving units, chillers and other fixtures.

#### Compliance with promotions rules

Moving confectionery away from till points was a particular challenge in small outlets, and some managers questioned whether it was reasonable to apply the same rules to shops with vastly different floor space and layouts. Most of the outlets, prior to HRS, offered ‘meal deals’ – promotional bundles offering a sandwich, snack (usually crisps, sometimes fruit) and drink for a fixed price. Managers perceived that the initial restriction of the snack item to fruit led to a drop in meal deal sales, while the subsequent amendment to permit baked crisps (see Table 1) revived customer interest in some outlets, although others perceived that meal deal sales continued to be below pre-HRS levels. Table 4 suggests that there was no increase overall in the use of price promotions for fruit at Wave 2, and this was reflected in the interviews, with some managers perceiving little demand for and high levels of wastage of fresh fruit, although a few found that fruit sales did well. A view was expressed by some managers that outlets needed to be more creative in devising alternative promotions to compensate for loss of sales from confectionery promotions, such as offers on non-food products, and several queried why HRS rules did not permit promotions on ‘healthier’ snacks such as cereal bars.

#### Factors affecting implementation

Differences in ease of implementation of the HRS emerged between small independent retailers and retailers who were part of a large retail group or national charity, with the latter benefitting from centralised processes for sourcing of new products, planograms, briefing materials and training. Being part of a retail group or national charity enabled retailer managers to learn from other managers in the group, particularly those who had implemented HRS earlier. However, centralised processes were sometimes seen as not sufficiently flexible to cope with individual shop characteristics and contexts, with some managers feeling constrained and that their local knowledge was underutilised. Hospital shops in Scotland formed only a small proportion of the parent organisation’s entire estate (particularly the case with national retailers), which meant that head office staff took some time to familiarise themselves with HRS requirements. In contrast, independent retailers identified limited resources and lack of knowledge as barriers to implementing HRS. Achieving compliance tended to be a lengthy process in these shops, with managers having to source new products and master all the new criteria and processes largely unsupported.

Across all of the sample, there was a feeling that more support and guidance could have been provided. Firstly, the evolving nature of the HRS criteria caused frustration. Managers perceived that some products which had initially been compliant with nutrition criteria were later reclassified as non-compliant, and vice versa; clarifying what was permitted and keeping up with the changes consumed time and effort. Secondly, there was a feeling that retailers could have been provided with more practical assistance, such as lists of specific compliant products and suppliers. Thirdly, although most managers valued feedback received during visits and inspections from the SGF, some felt that guidance on improvement was lacking: ‘It was like, “you failed”. Well, what else are we supposed to do? We need some advice then.’ (Outlet 7, Wave 2). Finally, managers felt that more could have been done by the NHS to explain to customers why HRS had been introduced. Overall, customer response was generally described as muted, but some complaints were received, primarily from NHS staff who were regular customers.

#### Perceptions of the impact of HRS: threats and opportunities

Managers varied in their support for and expectations regarding the impact of HRS. The national charity expressed strong support for HRS and took the opportunity to rebrand and redesign all its hospital outlets as offering healthy choices; in interviews, managers were reasonably optimistic that outlets would continue to be viable and attractive to customers. In contrast, managers of other outlets were initially ambivalent about HRS and somewhat pessimistic about the potential negative impact on sales and profits, and approached implementation seeking the best way to mitigate anticipated losses. By Wave 2, when all outlets had achieved compliance and changes had bedded in, implementation-related concerns tended to have abated.

By Wave 2, some managers were beginning to identify potential opportunities in HRS, such as the unanticipated popularity of bottled water and sugar-free confectionery. Managers of not-for-profit outlets perceived that HRS helped to some extent to ‘level the playing field’ between themselves and large commercial outlets, which had previously been able to offer a much wider range of confectionery on promotion. There was a perception that the demand for healthier products driven by HRS had led to positive changes further up the supply chain, in the shape of increased choice in the wholesale sector, and suppliers modifying their ingredients (for example, one large supplier of sandwiches increased its healthier options in response to the HRS requirements). Some positive impacts for smaller suppliers of more niche products were noted: one manager described how a local shop which stocked sugar-free confectionery lines had received ‘an absolute boost to her business….at least half a dozen hospitals buying from her’ (Outlet 5, Wave 2).

## Discussion

The Healthcare Retail Standard is an innovative mandatory approach to making the food retail environment healthier. As far as we are aware, it is the only national mandatory standard internationally which both rebalances the product range and restricts the use of promotions. By creating a consistent approach across hospital shops in Scotland, HRS changed the context in which food purchase decisions are made by healthcare staff, visitors and patients. Healthcare staff comprised the largest customer group and can be seen as the main policy beneficiaries; this is particularly important given that working adults consume a substantial amount of daily energy intake at work [29], that workplace food exposures may be less healthy than those at home [30], and that there is growing concern about diet and obesity in healthcare staff [17, 18]. However, hospital shops are also used by patients and visitors, and have been recognised as potential determinants of dietary behaviour and obesity in the general population including children [31].

Focusing in particular on two product categories, chocolate and fruit, HRS resulted in a substantial reduction in both the number of chocolate products on display and in the use of promotions for chocolate, although we did not observe a directly corresponding increase in the number of fruit products or promotions, reflecting in part retail managers’ perceptions of low customer demand. However, managers were in some cases surprised when there proved to be a customer demand for other healthier products such as water, cereal bars and sugar-free confectionery. Research in other food retail settings has similarly suggested a tendency for customer interest in healthier products to exceed expectations following marketing interventions [32]. Furthermore, small but positive changes were observed to the supply chain, such as increased choice and modifications to ingredients. These findings suggest that schemes such as HRS can act as a stimulus to encourage both retailers and suppliers to have more confidence in customer interest in healthier choices and to experiment with new healthier permutations.

A key finding from the study is that different types of retail outlets – commercial, not-for-profit, retail groups and independents – were able to implement HRS. For the national retailers, their hospital shops were a small proportion of their total estate, which included high street stores across Scotland and the rest of the UK, as well as hospital shops in other countries of the UK which did not have an equivalent to HRS. Adhering to HRS required these retailers to adjust their highly centralised processes. While this was challenging, the study demonstrates that, through regulatory schemes such as HRS, it is possible to bring about change in the retail sector. Importantly, implementation of HRS across all hospital shops created a level playing field between all the different types of retailers in the hospital sector, and meant that there was no opportunity for customers to go elsewhere for unhealthy promotions, other than outside the hospital entirely. As few of the hospitals had many external retail competitors within the immediate vicinity of the hospital [27], this meant that HRS had the potential to impact positively on customer purchasing behaviour. Given the disparity more generally between different types of food retail outlets in terms of their ‘healthfulness’ [33], policies which have the potential to create consistent and convenient offerings are to be welcomed.

Our findings contribute to the ongoing debate about the merits of voluntary versus mandatory approaches to improving the healthiness of the food retail environment [34, 35] Although some voluntary and self-regulatory initiatives have produced positive results (notably reductions in salt intake [36], these often fail to reach targets set out by government and can suffer from the lack of a consistent inspection and enforcement regime, resulting in variable or short-lived implementation [35]. This was not a problem in the first year of HRS, with all hospital shops being inspected and assessed for compliance, and compliance being a condition of the NHS contract with the retail business. HRS is consistent with a growing call for mandatory approaches to the modification of the food retail environment [5]. The UK Soft Drinks Industry Levy [37], which aims to encourage reformulation of sweetened soft drinks by compelling manufacturers to pay a levy linked to sugar content per 100 ml, is one such example. Recent work on salt intake [38] points to a comprehensive package of measures involving multiple components and population-wide policies such as mandatory reformulation in order to impact significantly on sodium intake.

A number of areas for improvement and future action were identified which have relevance both for HRS and for the development of similar schemes in other contexts. Firstly, more support and guidance could have been offered during implementation, particularly for independent retail outlets which could not access help from a larger host organisation. Conducting a pilot scheme to test and finalise the criteria before roll-out could have avoided the situation whereby criteria were evolving as retailers were attempting to implement. Positive messages to customers publicising and endorsing the new healthier retail offerings could have built interest, reduced the likelihood of complaints, and underlined the NHS’s commitment to employee wellbeing. The limited use of fruit promotions suggests that more focus could have been put on helping retailers to identify and implement positive opportunities associated with HRS, building on evidence that price promotions can increase purchasing of healthier foods, especially when combined with other types of promotion such as product information [39].

The study had a number of strengths and limitations. Mixed methods enabled us to examine the implementation of HRS from a variety of perspectives. Observational data provided objective information on changes to the product range and promotions, while qualitative interviews provided detailed insight into how the rules were interpreted, the challenges faced in implementation and any opportunities identified by retail managers. In terms of study limitations, the sample size, 13 shops and 4 trolley services, was small, although it was representative of the diversity across the hospital retail environment and included nearly one fifth of all shops and a tenth of all trolley services. The small number of trolley services in the audit and variability in when the trolleys were observed means that the results should be treated with caution. By the time the study was commissioned, some retail outlets had already begun to implement aspects of HRS, meaning that the Wave 1 data do not provide a pre-implementation baseline; consequently the changes associated with HRS implementation may have been of greater magnitude than our data show. Sales data were not available to the research team, so we could not verify managers’ comments regarding impact on sales or accounts of which products had increased/decreased sales.

The study flags up a number of implications for future research and action. Regarding HRS specifically, future research could examine impact on retail outlets’ financial viability and profits, as well as examining customer response in terms of any changes in frequency of visiting hospital shops, level of spend and products purchased. Potential positive changes to the food supply chain and product reformulation associated also merit further investigation. Healthier promotions could be developed and tested. Long term monitoring of such schemes is needed to assess whether they are financially sustainable for retailers [40] and capable of delivering increases in healthy purchasing by customers without compromising profits [41]. Consideration should be given to whether the principles behind HRS could be extended to rebalance promotional and provisioning activities between healthy and unhealthy products in the food retail sector more broadly [5]. HRS provides an exemplar regulatory template which has the potential to be developed and tested in settings such as other workplaces, education, military, prison) where HRS-type requirements and compliance could be built into contracts or local licensing systems.

## Conclusions

Positive changes in food retail outlets occurred after hospital shops were required to implement HRS, an innovative set of mandatory criteria which rebalanced the product range and restricted the use of promotions for less healthy foods. By creating a consistent approach across hospital shops in Scotland, HRS changed the context in which food purchase decisions are made by hospital staff, visitors and patients. It provides a regulatory template and implementation learning points for influencing retail environments in other jurisdictions and settings.

## Supplementary information

**Additional file 1.** Retailer interview guide.

**Additional file 2.** Observation Protocol Fixed Outlets Wave 1.

**Additional file 3.** Observation Protocol Fixed Outlets Wave 2.

**Additional file 4.** Observation Protocol Trolley Services Wave 1.

**Additional file 5.** Observation Protocol Trolley Services Wave 2.

## Data Availability

The datasets used and/or analysed during the current study are available from the corresponding author on reasonable request.

## Abbreviations

HRS: Health Retail Standard
NHS: National Health Service
PMP: Price Marked Pack
SD: Standard Deviation
SGF: Scottish Grocers Federation
SKU: Stock Keeping Unit

## Footnotes

1 We use the terms ‘commercial’ and ‘not-for-profit’ to refer to these two categories in the rest of the paper, although it should be noted that most of those in the not-for-profit category did nonetheless aim to maximise sales in order to generate revenue for charitable activities, and also employed paid staff as well as volunteers.
