# Supplementary material for: Making hospital shops healthier: evaluating the implementation of a mandatory standard for limiting food products and promotions in hospital retail outlets
Source: BMC Public Health. 2020 Jan 30;20:132. doi: 10.1186/s12889-020-8242-7 (PMC6990565; doi:10.1186/s12889-020-8242-7)
Supplement: Supplementary file 1 — Additional file 1. Retailer interview guide. [file 12889_2020_8242_MOESM1_ESM.docx]

**Scottish Healthcare Retail Standard Study**

**Retailer Interview Guide**

## A. Introduction

## Recap purpose of study

- Check you have sufficient time (approx. 40 mins) and privacy
- Reiterate confidentiality and voluntary nature
- Check participant is happy for the interview to be recorded and explain its purpose
- Check participant is happy for the researcher to complete a short observation of in-store displays and to take some photographs of the shop layout after the interview
- Check if the participant has any questions before proceeding

The following questions should NOT be regarded as prescriptive but rather as examples of probes for initiating discussion around key research themes. Interviews should be conducted in a naturalistic manner. Themes need not necessarily be explored in the following order and should be tailored in accordance with participant’s position and experience.

**B. Interviewee and case business** (limit follow-ups to ‘any changes since we last spoke’):

1. Confirm position - owner, shop manager, nominated representative etc
2. Overview of business ownership (including whether external lease or in-house NHS service), management arrangements (including whether voluntary or commercial) and range of services provided, including trolley services, market stalls and fruit barras
3. Overview of key business areas in terms of turn-over and customer groups
4. Overview of competitive context: other businesses on and off site, including those onsite also covered by the Standard and variation in implementation schedules.

**C. Awareness, understanding and attitudes towards HRS:**

1. Awareness and understanding of the aims and purpose of the Standard (explore perceived importance of food choice to health) / check if already comply with the Healthy Living Award – HLA (mixed retail and catering only)
2. Awareness and understanding of the qualification criteria and implementation timetable – what are the qualifying criteria and when does the standard apply/need to be enacted? / (if appropriate)
3. Awareness and understanding of the scope of the Standard – what aspects of food provision and marketing does it cover? / how easy/difficult have you found it to understand?
4. What have been the main sources of advice and information on the Standard and its implementation (if any)? / how proactive have you been in seeking information? / what agencies have provided you with information? (Probe for external agencies including SGF guidance and local Health Board as well as support from within the retail group and other retailers as appropriate. Request copies of any documents used to support implementation such as planograms and guidance documents)
5. Attitudes and level of support for the Standard, esp. regarding its perceived value and effectiveness – how do you feel about the Standard? / what’s you view on its (likely) impact and effectiveness? / have your views changed in any way overtime/having implemented the Standard? (follow-up only)

**D. Implementation of HRS:**

1. What actions do you intend to take / have you taken in order to prepare for / implement the Standard? Will this / has this involve(ed) any staff training or briefing? (Request retailer to walk you through the store to highlight the physical changes planned and made, esp. regarding number of products on sale, display arrangements and layouts, and product promotions)
2. Do you expect it will / has it affect(ed) the products that you stock in any way / your product range in anyway? (Probe for any changes to key product categories: fruit, vegetables, chocolate, soft drinks, meal items, ‘healthy items’ including nuts and cereal bars)
3. What do you expected to be / have been the main challenges to implementing the Standard (if any) / what elements of the Standard do you anticipate will be / have been easier/more difficult to implement? how do you intend to /have you address(ed) these issues?
4. What do you expected to be / have been the main opportunities presented by the Standard (if any) and how do you intend to /have you taken advantage of these? (Probe for any impact of change in product lines and promotions to business image and customer profiles and purchase patterns – see under impact below)
5. Do you anticipate / have any other local businesses been affected by the Standard? In what way / how will/have they respond(ed)? Will/has it had any implications for your own business? (Probe for any variations in how other businesses have responded and been effected - see also competitive impact below)
6. What arrangements are in place for monitoring compliance with the Standard? / (if appropriate) are you aware if any efforts have been made to check compliance? / what kind of feedback have you received if any / how helpful has this been? (Probe for interactions with SGF and local Health Board)
7. What are the implications for you as a business for failing to comply or for any short comings in compliance, including sanctions and impact on lease renewal?

**E. Expected and perceived impact of HRS:**

1. Do you anticipate the Standard will have / has had any impact on your business performance, including impact on turnover, profits, long term viability, (Probe for any changes in more/less profitable food lines)
2. Do you anticipate / has there been any costs to the business associated with making the necessary changes to accommodate the Standard? (Probe for installation of chiller shelves, level of food wastage) / has the Standard allowed you to make any savings? / (if appropriate) has it affected the value and attractiveness of lease agreements, willingness to renew lease agreements?
3. Do you anticipate the Standard will have / has had any impact on competitor environment / your ability to compete with other local businesses / has had a differential impact on your local competitors? (Probe for how the Standard will/has effect(ed) other local businesses and any actions anticipated/taken, including information on their implementation schedule / intentions)
4. Do you anticipate the Standard will have / has had any impact on customer numbers / profiles / loyalty / purchasing patterns / average spend? (Probe for any changes in type of customer, esp. perceived affluence, amount available to spend, shifts in popularity of types of food purchased re healthy and less healthy choices esp. fruit and chocolate, and shifts in motives for purchase esp. more/less customers buying meal choices)
5. How do you expect customers will respond / have responded to the Standard if at all? (Probe for level and nature of feedback, including any awareness of and attitudes towards the Standard and/or specific changes made in order to comply with the Standard)
6. Do you anticipate the Standard will have / has had any (other) unintended consequences, positive or negative?

**F. Trolley service**

(If the retailer also operates a trolley service in the hospital ask the following key questions. These are intended to cover the same broad areas)

1. What are your feelings about the Standard also applying to products offered as part of the hospital trolley service?
2. How difficult or straightforward do you anticipate it will be/has it been to implement in the trolley service?
3. What kind of changes do you expect to make /have you made to accommodate the Standard?
4. What do you anticipate will be /have been the main challenges? / how does this compare with the challenges in the shop setting?
5. What support and guidance have you received if any?
6. What do you anticipate will be /has been the financial impact?
7. How will it / has it impact(ed) on customers?

**G. Introduce the observation:**

Confirm that retailer is happy for researcher to conduct observation. Probe the following as appropriate to assist in completing the observation form:

- Sales floor area (metres square)
- Opening times (inc any day variations)
- Details of any promotions not able to be observed (eg. verbal promotions at the till)
- Whether a planogram is used to guide layout

(Where appropriate) clarify details about trolley service:

- - How many trolleys operated
  - What times do they operate (inc any day variations)
  - What hospital areas does the trolley service cover (probe using protocol pre-coded list)
  - Check opportunities to observe trolley set-up and layout.

**H. Next stages, admin and close:**

- Baseline interviews only:
  - Confirm interest in and likely timing for follow-up visit
- Offer incentive and obtain receipt
- Thank and close
