# Supplementary material for: Making hospital shops healthier: evaluating the implementation of a mandatory standard for limiting food products and promotions in hospital retail outlets
Source: BMC Public Health. 2020 Jan 30;20:132. doi: 10.1186/s12889-020-8242-7 (PMC6990565; doi:10.1186/s12889-020-8242-7)
Supplement: Supplementary file 5 — Additional file 5. Observation Protocol Trolley Services Wave 2. [file 12889_2020_8242_MOESM5_ESM.docx]

| **ID** |  |
| --- | --- |

**Wave 2 observation protocol: Trolley services**

**Observer(s):**

……………………………………………………………………………………………………………

**Day, date and time of visit:**

……………………………………………………………………………………………………………

**Outlet name**:

……………………………………………………….…………………………………………………

**Location** (hospital & health board area):

…………………………………………….…………………………………………………………….

**Opening hours** (inc any variations at weekends etc):

…………………………………………….…………………………………………………………….

**Q1. Outlet category**

**a) Management category**

- Externally managed: multiple
- Externally managed: symbol group
- Externally managed: non-affiliated independent
- Voluntary
- Other: Write in:……………………………………………………………………..

**b) Retail category**

- 1. Newsagent/gift (eg. newspapers, gifts, confectionery, drinks)
- 2. Convenience (eg. snacks, grocery, sandwiches, salads, drinks)
- 3. Retail other (write in):

…………………………………………………………………………………….

- 4. Mixed retail & catering (eg. includes takeaway hot food), assessed as predominantly retail

**Q2. Scale of trolley service**

How many trolleys operate in the hospital? Write in: ……………………………………………………..

**Q3. Route**

Which areas of the hospital are visited by the trolley service? Tick all that apply

- In-patient wards
- Outpatient depts including waiting areas
- Staff rest areas/common rooms
- Staff offices/work areas
- Staff residences
- Other: write in:………………………………………………………………….

**Q4. Appearance of trolley**

Using photographs where possible, produce a diagram of the trolley layout, including top, sides and any pull-out drawers/trays.

SHADE all areas which display chocolate and all areas which display fruit (use two different colours).

|  |
| --- |

**Q5. Does the trolley sell chocolate (see definition)?**

**Chocolate products are:**

Chocolate blocks: Solid blocks of chocolate incl. milk, plain & white, of all sizes. Includes blocks with added ingredients, such as fruit and nuts. Includes eggs.

Countlines: Products that contain chocolate as main ingredient, as well as other ingredients such as caramel, fruit, wafer & biscuit. Eg. Mars bars, Snickers, Twix and Crunchie.

Selflines: Bags, roll-wraps & tubes of all sizes, containing individual pieces of product coated in chocolate. Eg. Smarties, Maltesers, Revels and Munchies.

EXCLUDE:

- Chocolate ice cream
- Chocolate biscuit such as McVitie’s/Cadbury Chocolate Digestives, where chocolate is not major ingredient/where marketed as biscuits.
- Confectionery items based mainly on sugar
- Cereal bars
- Cakes and desserts
- Yes
- No

**Q6. How many different chocolate**

**products/ shop keeping units (SKUs) are on display?**

Write number in each display area on diagram at Q3.

Count different sizes and flavours of

same brand variant as distinct units.

Total number:…………………………………………………………

Record any products you are uncertain about (inc. photos):

…………………………………………………………………………

………………………………………………………………………….

**Q7. How many of the following types of promotions
for chocolate are observed?**

(*take photographs if possible*):

| Type of promotion | **Number** |
| --- | --- |
| Promotional product displays  (incl. branded units, merchandising strips) |  |
| Price-marked packs  (price printed on packaging or outer wrapper) |  |
| Multi-buys and quantity/bundle discounts (incl. “3 for 2”, “3+1 free”, reduced price chocolate bar with newspaper) |  |
| Advertising materials  (incl. shelf signage, shelf danglers, posters etc). |  |
| Other: write in type (include verbal offers by trolley staff) |  |

**Q8. Does the trolley sell fruit (see definition):**

**Fruit products are:**

Fresh fruit: Sold loose or pre-packed

Fresh fruit salad/fruit pots

Exclude:

Dried fruit bags or loose

Tinned fruit

Chocolate coated fruit

Dried fruit and nut mixes

- Yes
- No

**Q9. How many different fruit**

**products/SKUs are on display?**

Write number in each display area on diagram at Q3.

Count different quantities (eg. loose and pre-packed) as separate units, also count different varieties as separate units.

Total number:…………………………………………………………

**Q10. How many of the following types of promotions for fruit are observed?**

(*take photographs if possible*):

| Type of promotion | **Number** |
| --- | --- |
| Promotional product displays  (incl. branded units, merchandising strips) |  |
| Price-marked packs  (price printed on packaging or outer wrapper) |  |
| Multibuys and quantity/bundle discounts (incl. “3 for 2”, “3+1 free”, reduced price fruit bar with newspaper) |  |
| Advertising materials  (incl. shelf signage, shelf danglers, posters etc). |  |
| Other: write in type (include verbal offers by trolley staff) |  |

**Q11. Quality/appeal of fresh fruit**

Please photograph and rate the quality/appeal of the fresh fruit on display:

| Most/all of the fruit  looks appealing/fresh |  |  |  | Most/all of the fruit looks unappealing/  dried out/rotten |
| --- | --- | --- | --- | --- |
| 1 | 2 | 3 | 4 | 5 |

**Q12. Standard food offer: meal deal availability**

**Does the trolley offer a meal deal (eg. bundle discount on sandwich/other meal item plus other items)?**

- Yes: write in price and details (take photograph if possible)

……………………………………………………………………………………………..

……………………………………………………………………………………………..

……………………………………………………………………………………………..

- No, sells sandwiches/other meal items, snacks and drinks individually
- No, does not sell sandwiches/other meal items

**Q13. Cost of individual items in a standard food offer**

Please write in the prices of the following items, if sold.

If multiple items at cheapest/most expensive price, record details for all.

Take photographs of products showing FOP labelling if possible.

| Item | Description | Price | Not sold |
| --- | --- | --- | --- |
| a. Cheapest roll/ sandwich/ wrap/ baguette | Include brand, filling, dressing/sauce, type of bread |  |  |
| b. Most expensive roll/ sandwich/ wrap/ baguette |  |  |  |
| c. Cheapest 500ml bottled pure water | Include brand, still/sparkling. |  |  |
| d. Most expensive 500ml bottled pure water |  |  |  |
| e. Cheapest 330ml soft drink excl, pure water | Include brand, flavour, any other info |  |  |
| f. Most expensive 330ml soft drink excl. pure water |  |  |  |

**Q14. Competing mobile outlets on study site**

Describe any other competing mobile retail outlets that operate within the hospital site:

………………………………………………………………………………………………………………………………………………………………………

………………………………………………………………………………………………………………………………………………………………………

………………………………………………………………………………………………………………………………………………………………………

……………………………………………………………………………………………………………………………………………………………………....
